# Papillary thyroid cancer coexisting with thyroid tuberculosis: A case report

**DOI:** 10.3892/ol.2014.1901

**Published:** 2014-02-21

**Authors:** LIWEI MENG, SHAN HU, LIMING HUANG, CHAOYANG XU

**Affiliations:** Department of Breast and Thyroid Surgery, Shaoxing People’s Hospital, Shaoxing Hospital of Zhejiang University, Shaoxing, Zhejiang 312000, P.R. China

**Keywords:** papillary thyroid cancer, thyroid tuberculosis, thyroidectomy

## Abstract

The current study presents the case of a 56-year-old female, with thyroid nodules showing signs of malignancy under B-mode ultrasonography, who was admitted to Shaoxing People’s Hospital. A histopathological examination revealed the coexistence of papillary thyroid cancer (PTC) and thyroid tuberculosis (TB). A total thyroidectomy and selective neck dissection (left side, levels III, IV and VI) were performed. This report presents a rare case showing the malignant growth of thyroid nodules and the development of thyroid TB, which implicates the possible role of mycobacterial infection in the tumorigenesis of PTC.

## Introduction

Tuberculosis (TB) is a global threat to public health. The World Health Organization reported that in 2011, approximately two billion individuals were known to be infected with *Mycobacterium tuberculosis* and 1.4 million deaths were associated with TB (1). TB may be categorized into pulmonary and extrapulmonary TB. Extrapulmonary TB comprises 20% of all TB cases and occurs in a variety of organs (2). TB of the thyroid gland is an extremely rare disease.

Cancer is also a global threat and the second leading cause of mortality following coronary artery disease, with 7.6 million deaths in 2008 (3). Thyroid cancer is a common type of cancer. In the United States, thyroid cancer comprises 1% of all cancers and accounts for 0.2% of cancer mortality (4). Papillary thyroid cancer (PTC) is the most common well-differentiated cancer of the thyroid gland and accounts for 80–85% of well-differentiated thyroid cancers (5).

The coexistence of malignant lesions and TB at the same anatomical location in a patient is extremely rare, but has been reported in various organs (6). However, only three cases in which thyroid TB occurred concomitantly with thyroid cancer have been reported (7–9). Among them, two cases of thyroid TB were found post-operatively and one case of thyroid TB was identified in the fine-needle aspiration cytology (FNAC). The present study reports a case of coexisting TB and PTC diagnosed by the histopathological examination of a resected thyroid specimen. The patient provided written informed consent.

## Case report

A 56-year-old female was admitted to the Department of Breast and Thyroid Surgery (Shaoxing People’s Hospital, Shaoxing, Zheijiang, China) in August 2012 due to the presence of thyroid nodules on B-ultrasound. The patient first became aware of these thyroid nodules during a regular health examination in 1997. The nodules developed slowly and one of the nodules showed signs of malignancy in the most recent B-mode ultrasound examination. The patient had no history of pulmonary or extrapulmonary TB or any known contact with any individuals with TB.

**Figure 1:**
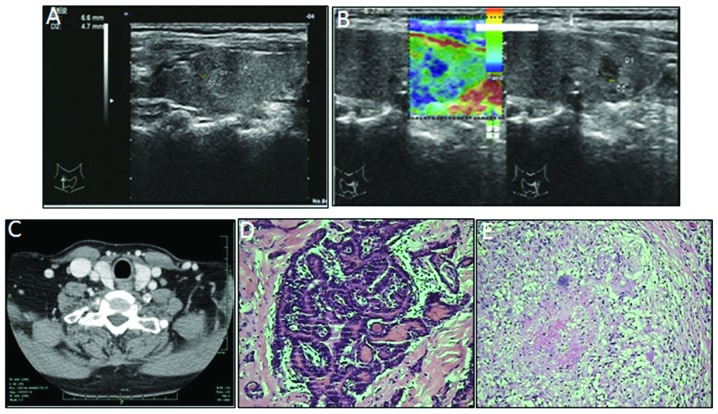
Images and histological analysis of the thyroid. (A) B-mode ultrasonography showing hypoechoic nodules and (B) one nodule with sonographic characteristics of a thyroid malignant nodule. (C) Computed tomography of the neck showing enlarged cervical lymphonodules on the left side. (D) Histological characteristics of papillary cancer. Typical papillary structures composed of a fibrovascular core and lined by enlarged epithelial cells. (E) Histological characteristics of thyroid TB showing epitheloid cells, lymphocytes and giant cells of Langerhans type, with necrotic caseation.

A clinical examination revealed a first to second degree multinodular goiter, which was mobile, soft and not painful on palpation. There was no palpable lymphadenopathy in the neck. Thyroid function tests showed a hypothyroidism state: Increased thyrotropic-stimulating hormone [TSH; 6.12 μIU/ml; normal range (NR), 0.35–5.5 μIU/ml] and antithyroglobulin antibody levels (27.8 IU/ml; NR, ≤4.11 IU/ml); decreased free triiodothyronine (2.96 pmol/l; NR, 3.0–6.51 pmol/l) and triiodothyronine (0.9 nmol/l; NR, 0.92–2.79 nmol/l) concentrations; normal thyroxine and free thyroxine, thyroglobulin and thyroid-binding globulin levels; and a negative result for antithyroperoxidase antibody. Biochemical analyses only showed elevated C-reactive protein levels (31.44 mg/l; NR, ≤8 mg/l). Thyroid B-mode ultrasonographyrevealed a number of hypoechoic nodules in the bilateral lobe of the thyroid gland, with sizes varying between 5 and 11 mm. However, one of these nodules (7–8 mm in size) located near the top of the left thyroid lobe was a heterogeneous hypoechoic lesion, and infiltrative margins with microcalcifications were identified by ultrasonography (Fig. 1). B-mode ultrasonography showed a malignant nodule and cervical lymphadenectasis. Computed tomography revealed that the cervical lymphonodule was enlarged on the left side, at levels III and IV (Fig. 1). The chest X-ray was normal and other extrapulmonary TBs were excluded.

A total thyroidectomy and selective neck dissection (left side, levels III, IV and VI) were performed. The lobes were opened and certain areas appeared to be colloid-rich and necrotic. A hard, whitish lesion 6 mm in diameter, which was not clearly separated from the normal tissue, was identified in the left lobe. The extracted lymph nodes were soft, oval and 10 mm in diameter. The intraoperative histological evaluation revealed thyroiditis and PTC. The final diagnosis was established by definitive histopathological examination. The histopathological examination of the dissected cervical lymph nodes showed the presence of TB, but no metastasis. Following surgery, a tuberculin skin test (PPD test) showed strong positivity. The final diagnosis was of PTC with thyroid TB (Fig. 1) and tuberculous lymphadenitis. There were no post-operative complications. The patient was treated with isoniazid, rifampicin and ethambutol for six months, prior to a permanent substitution with L-thyroxin (100 μg per day). During the subsequent regular follow-up examinations there were no signs of disease recurrence.

## Discussion

Thyroid gland TB is a rare disease with a frequency of 0.1–0.4% in histologically diagnosed thyroid specimens (10). In Asian countries, such as India and Turkey, where the prevalence of TB is high, the incidence of thyroid gland TB remains low (0.6–1.15%) (11,12). Thyroid TB is difficult to diagnose in the clinic due to a lack of specific signs and symptoms. Thyroid TB may present itself as an isolated nodule, diffuse legion or multinodular goiter, and even as an abscess or chronic skin sinus (10). Imaging techniques are not very useful in forming a diagnosis (13). FNAC with acid-fast bacilli is considered the best method for the diagnosis of TB pre-operatively (14). However, the majority of thyroid TB cases are diagnosed based solely on the post-operative histopathological examination. In the present case, the identification of TB in cervical lymphadenopathy and a strongly positive PPD test supported the diagnosis of TB. The final diagnosis was established by the definitive histopathological examination of a resected specimen

In the present case, hypothyroidism was observed, which is uncommon for thyroid TB or PTC. This observation may have been associated with the long disease history (16 years) of the thyroid TB, where fibrotic tissue may have replaced normal thyroid follicles. A previous study suggested the use of antituberculous agents combined with surgical removal of the affected area of the thyroid gland or surgical drainage to treat thyroid TB (15). A total thyroidectomy was not indicated due to consequent hypothyroidism. In the present case, a total thyroidectomy was performed due to the coexistence of PTC.

Generally, the appropriate microenvironment for malignant development may be created by chronic inflammatory conditions using numerous mechanisms (16). Mycobacterial infections establish chronic and persistent inflammation. Previous studies have reported that mycobacterial cell wall components are capable of inducing DNA damage through the production of nitric oxide and reactive oxygen species (17). This DNA damage has been implicated in inflammation-related carcinogenesis (18). *Mycobacterium tuberculosis* has also been found to induce antiapoptotic activity through the upregulation of B-cell lymphoma 2 gene expression (19). Moreover, certain clinical and experimental studies have observed elevated concentrations of prostaglandins following mycobacterial infection (20). The combination of direct DNA damage, apoptosis inhibition and chronic inflammation may result in a microenvironment that is highly favorable for tumorigenesis (17). In the present case, the histopathological findings revealed typical inflammatory characteristics of thyroid TB. Thus, the development of PTC may be associated with TB infection. Furthermore, the high TSH levels caused by disruption of the thyroid tissue promoted tumor cell growth. In addition, the presence of TB lymphadenitis may result in overstaging when using the tumor-node-metastasis system. Pre-operative lymphatic puncture effectively prevents overstaging and excessive surgery.

In conclusion, this study reported a rare case of coexisting PTC and thyroid TB, and implicated the possible role of mycobacterial infections in the tumorigenesis of PTC.
